# Supplementary material for: Benchmarking large language models for biomedical natural language processing applications and recommendations
Source: Nat Commun. 2025 Apr 6;16:3280. doi: 10.1038/s41467-025-56989-2 (PMC11972378; doi:10.1038/s41467-025-56989-2)
Supplement: Supplementary file 1 — Supplementary Information [file 41467_2025_56989_MOESM1_ESM.docx]

**Supplementary Material**

**S1. Prompt engineering**


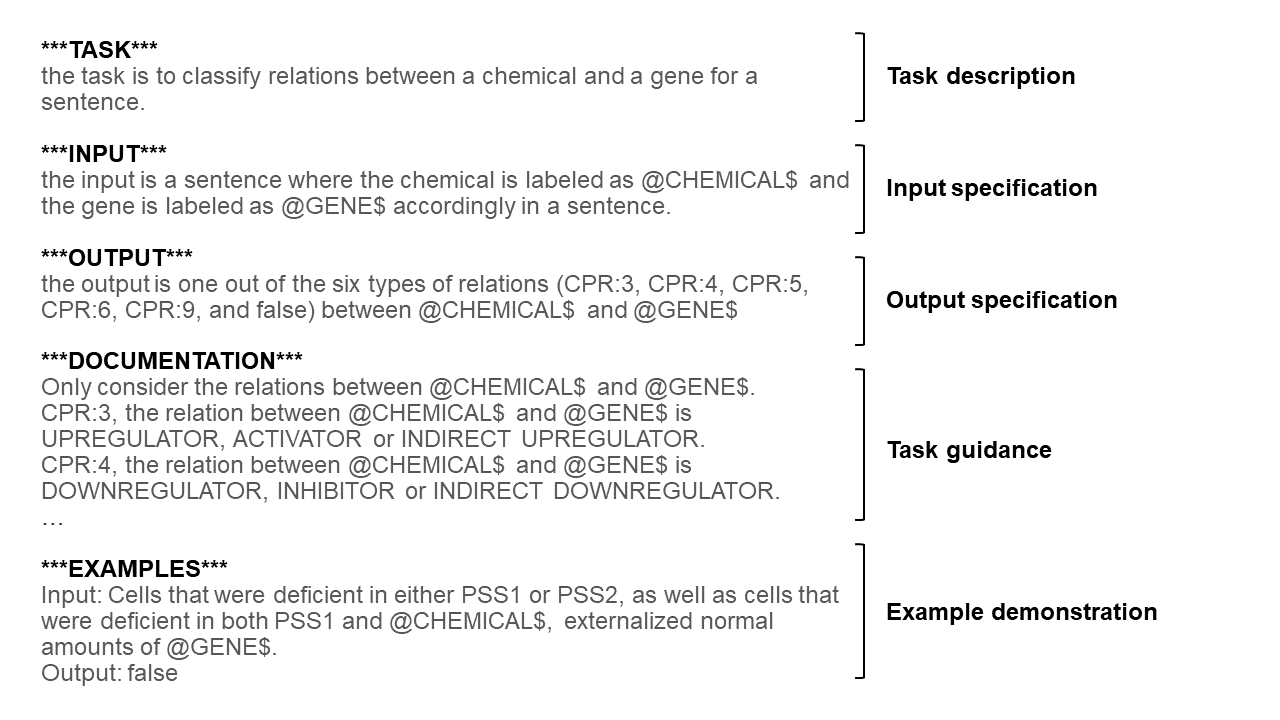


Figure S1. An annotated one-shot prompt example for biomedical relation extraction.

**S1.1 An annotated prompt example**

Figure S1 shows an example prompt for relation extraction in ChemProt. The prompt consists of (1) task descriptions (e.g., classifying relations), (2) input specifications (e.g., a sentence with labeled entities), (3) output specifications (e.g., the relation type), (4) task guidance (e.g., detailed descriptions on relation types), and (5) example demonstrations if an example is provided.

All the prompts used for the 12 benchmarks are also publicly available via <https://github.com/BIDS-Xu-Lab/Biomedical-NLP-Benchmarks>.

**S1.2 Parameters**

For zero- and few-shot approaches, we used a temperature of 0 to minimize variance for both GPT and LLaMA-based models. Additionally, for LLaMA models, we kept other parameters unchanged and set the maximum number of generated tokens (the max_new_tokens parameter) per task. The maximum number of generated tokens was 512, 20, 64, 128, 512, and 512 for named entity recognition, relation extraction, multi-label document classification, question answering, text summarization, and text simplification, respectively.

In addition, input truncation was applied to LLaMA models under the five-shot setting due to their maximum input length limit of 4096 tokens. We truncated the inputs for question answering, text summarization, and text simplification benchmarks. We first calculated the number of available tokens by subtracting the length of the prompt and input samples from its maximum input length. Then, we calculated the allowed length of tokens per shot (dividing by five). If the length of any shot exceeded the allowed length, we truncated it accordingly.

The related codes are also available via the repository.

**S2. Quantitative evaluation results**

**S2.1. Result reporting**

For quantitative evaluation, to quantify statistical significance between the LLM performance on the 12 datasets, we performed a two-tailed Wilcoxon rank-sum test with bootstrapping. We used a subsample size of 30 and repeated the process 100 times at a 95% confidence interval.

We employed the two-sided Wilcoxon rank-sum test to conduct comparative analyses between two lists of bootstrapped metrics from different LLMs. Specifically, we utilized the ranksums function from the scipy.stats package with parameters alternative='two-sided' and nan_policy='omit' for statistical analysis.

**S2.2 Primary and secondary evaluation metric results**

Table S1. Quantitative evaluations of the LLMs on the 12 benchmarks under zero-shot, one-shot, and fine-tuned settings. Both primary metric and secondary metric results are reported. State-of-the-art (SOTA) results, representing the reported best performance of studies using fine-tuned (domain-specific) language models before the LLMs and their backbone models, are also provided. The SOTA results are directly extracted from the studies. ^1^the study reported accuracy on MedQA (4-option); we applied the released model for inference on MedQA (5-option).

|  |  |  |  | | | **Zero/Few-shot** | | |  | | |  | |
| --- | --- | --- | --- | --- | --- | --- | --- | --- | --- | --- | --- | --- | --- |
|  |  | **SOTA results**  **before LLMs** | **Zero-shot** | | | **One-shot** | | | **Five-shot** | | | **Fine-tuned** | |
|  |  |  | **GPT-3.5** | **GPT-4** | **LLaMA 2 13B** | **GPT-3.5** | **GPT-4** | **LLaMA 2 13B** | **GPT-3.5** | **GPT-4** | **LLaMA 2 13B** | **LLaMA 2 13B** | **PMC LLaMA 13B** |
| **Named entity recognition** | | |  |  |  |  |  |  |  | | |  |  |
| BC5CDR-chemical | Entity F1 | 0.9500 | 0.6274 | **0.7993** | 0.3944 | 0.7133 | **0.8327** | 0.6276 | 0.7228 | **0.7979** | 0.5530 | **0.9149** | 0.9063 |
| NCBI Disease | Entity F1 | 0.9090 | 0.4060 | **0.5827** | 0.2211 | 0.4817 | **0.5988** | 0.3811 | 0.4309 | **0.6389** | 0.4847 | **0.8682** | 0.8353 |
| **Relation extraction** | | |  |  |  |  |  |  |  | | |  |  |
| ChemProt | Macro F1 | 0.7344 | 0.1345 | **0.3250** | 0.1392 | 0.1280 | **0.3391** | 0.0718 | 0.1758 | **0.3756** | 0.0967 | **0.4612** | 0.3111 |
|  | Micro F1 |  | 0.2376 | **0.3538** | 0.1232 | 0.2011 | **0.4109** | 0.0174 | 0.1864 | **0.4933** | 0.1554 | **0.8006** | 0.7659 |
| DDI2013 | Macro F1 | 0.7919 | 0.2004 | **0.2968** | 0.1305 | 0.2126 | **0.3312** | 0.1779 | 0.1706 | **0.3276** | 0.1663 | **0.6218** | 0.5700 |
|  | Micro F1 |  | **0.3862** | 0.3830 | 0.1650 | 0.2850 | **0.4819** | 0.4518 | 0.3202 | 0.4498 | **0.5938** | **0.8503** | 0.8438 |
| **Multi-label document classification** | | |  |  |  |  |  |  |  | | |  |  |
| HoC | Macro F1 | 0.8882 | 0.6722 | **0.7109** | 0.1285 | 0.6671 | **0.7093** | 0.3072 | 0.6994 | **0.7099** | 0.1797 | **0.6957** | 0.4221 |
|  | Micro F1 |  | 0.6605 | **0.7166** | 0.1495 | 0.6711 | **0.7205** | 0.3813 | 0.6992 | **0.7217** | 0.2229 | **0.6787** | 0.4536 |
| LitCovid | Macro F1 | 0.8921 | **0.5967** | 0.5883 | 0.3825 | **0.6009** | 0.5901 | 0.4808 | **0.6179** | 0.6077 | 0.3305 | **0.5725** | 0.4273 |
|  | Micro F1 |  | 0.6707 | **0.6809** | 0.5400 | 0.6656 | **0.6839** | 0.5997 | 0.6989 | **0.7202** | 0.4395 | **0.6668** | 0.5632 |
| **Question answering** | | |  |  |  |  |  |  |  | | |  |  |
| MedQA (5-Option) | Accuracy | 0.4195^1^ | 0.4988 | **0.7156** | 0.2522 | 0.5161 | **0.7439** | 0.2899 | 0.5208 | **0.7651** | 0.3504 | **0.4462** | 0.3975 |
|  | Macro F1 |  | 0.4096 | **0.5104** | 0.2226 | 0.4241 | **0.6171** | 0.2750 | 0.4307 | **0.6374** | 0.2875 | **0.4394** | 0.3932 |
| PubMedQA | Accuracy | 0.7340 | **0.6560** | 0.6280 | 0.5520 | 0.4600 | **0.7100** | 0.2660 | 0.6920 | **0.7580** | 0.6000 | **0.8040** | 0.7680 |
|  | Macro F1 |  | **0.4648** | 0.4604 | 0.2959 | 0.3761 | **0.5692** | 0.1916 | 0.5644 | **0.5872** | 0.5268 | **0.5628** | 0.5358 |
| **Text summarization** | | |  |  |  |  |  |  |  | | |  |  |
| PubMed | Rouge-L | 0.4316 | 0.2274 | **0.2419** | 0.1190 | 0.2351 | **0.2427** | 0.0989 | 0.2423 | **0.2444** | 0.1629 | **0.1857** | 0.1684 |
|  | BERT score |  | 0.7195 | **0.7242** | 0.6546 | 0.7227 | **0.7231** | 0.6467 | **0.7258** | 0.7253 | 0.6788 | **0.6623** | 0.6542 |
|  | BART score |  | -4.8334 | -4.8375 | **-4.7504** | **-4.8114** | -4.8548 | -5.8035 | **-4.7899** | -4.8488 | -4.8409 | -4.9772 | **-4.9212** |
| MS^2 | Rouge-L | 0.2080 | 0.0889 | **0.1224** | 0.0948 | 0.1132 | **0.1248** | 0.0320 | 0.1013 | **0.1218** | 0.1205 | **0.0934** | 0.0059 |
|  | BERT score |  | 0.6652 | **0.6904** | 0.6431 | 0.6775 | **0.6907** | 0.5921 | 0.6685 | **0.6858** | 0.6743 | **0.6265** | 0.0275 |
|  | BART score |  | **-5.0578** | -5.1767 | -5.2804 | **-5.1830** | -5.1909 | -5.9733 | **-5.1790** | -5.3470 | -5.5624 | **-5.4586** | -6.7826 |
| **Text simplification** | | |  |  |  |  |  |  |  | | |  |  |
| Cochrane | Rouge-L | 0.4476 | 0.2365 | **0.2375** | 0.2081 | **0.2447** | 0.2385 | 0.2207 | **0.2470** | 0.2469 | 0.2283 | 0.2355 | **0.2370** |
|  | FKG |  | 11.8815 | 11.0179 | **12.4385** | **12.3490** | 11.6752 | 12.2585 | **12.5152** | 11.7417 | 12.4712 | **11.8200** | 11.0660 |
|  | DCR |  | **10.1221** | 9.5580 | 9.8786 | **10.1796** | 10.0239 | 9.8024 | **10.1400** | 9.7333 | 9.8197 | **9.8072** | 9.7508 |
| PLOS | Rouge-L | 0.4368 | **0.2323** | 0.2253 | 0.2121 | **0.2449** | 0.2386 | 0.1836 | **0.2416** | 0.2409 | 0.1656 | **0.2583** | 0.2577 |
|  | FKG |  | 12.5182 | 11.1895 | **13.1722** | 13.2913 | 12.0144 | **13.3804** | 13.1201 | 12.0487 | **16.1425** | **13.9517** | 13.6370 |
|  | DCR |  | **11.0496** | 9.9077 | 10.1079 | **10.8338** | 10.1151 | 9.6846 | **10.6676** | 10.0256 | 8.9770 | **10.6670** | 10.5341 |

Table S1 shows the detailed results of LLMs on the 12 benchmarks using primary and secondary evaluation metrics.

**S2.3 Performance mean, variance, and confidence intervals**

Table S2. Mean, variance, and confidence intervals (shown in brackets) of the results obtained using bootstrapping with a subsample size of 30 and repeated the process 100 times at a 95% confidence interval.

|  |  | **Zero/Few-shot** | | | | | | | | | | | | | | | | | | | | | | | | | | | | | | | | | **Fine-tuned** | | | | |
| --- | --- | --- | --- | --- | --- | --- | --- | --- | --- | --- | --- | --- | --- | --- | --- | --- | --- | --- | --- | --- | --- | --- | --- | --- | --- | --- | --- | --- | --- | --- | --- | --- | --- | --- | --- | --- | --- | --- | --- |
|  |  | **Zero-shot** | | | | | | | | | | **One-shot** | | | | | | | | | | | | **Five-shot** | | | | | | | | | | |  |  |  |  |  |
|  |  | **GPT-3.5** | | | **GPT-4** | | | **LLaMA2 13B** | | | | **GPT-3.5** | | | | **GPT-4** | | | | **LLaMA2 13B** | | | | **GPT-3.5** | | | | **GPT-4** | | | | **LLaMA2 13B** | | | **LLaMA2 13B** | | **PMC LLaMA 13B** | | |
| **Named entity recognition** | | |  | | |  | | | | |  | | | |  | | | |  | | | |  | | | |  | | | |  | | | |  |  | | | |
| BC5CDR-chemical | Entity F1 | 0.6537±0.0851 (0.5103, 0.8322) | | | 0.8046±0.0661 (0.6798, 0.9111) | | | 0.3888±0.1018 (0.1949, 0.5677) | | | | 0.7248±0.0911 (0.5587, 0.8806) | | | | 0.8362±0.0675 (0.7089, 0.9438) | | | | 0.6418±0.0992 (0.4573, 0.8271) | | | | 0.7225±0.0922 (0.5328, 0.8902) | | | | 0.8018±0.0758 (0.6532, 0.9303) | | | | 0.5577±0.1081 (0.3469, 0.7637) | | | 0.9093±0.0517 (0.7883, 0.9853) | | 0.9113±0.0481 (0.8000, 0.9952) | | |
| NCBI Disease | Entity F1 | 0.4147±0.0836 (0.2621, 0.5673) | | | 0.5773±0.1060 (0.3642, 0.7420) | | | 0.2212±0.0959 (0.0440, 0.3967) | | | | 0.4823±0.0841 (0.3199, 0.6379) | | | | 0.6098±0.1014 (0.4035, 0.7767) | | | | 0.3834±0.0818 (0.2581, 0.5415) | | | | 0.4423±0.0842 (0.2831, 0.6131) | | | | 0.6480±0.0872 (0.4755, 0.8210) | | | | 0.4746±0.0761 (0.3145, 0.6190) | | | 0.8573±0.0568 (0.7138, 0.9578) | | 0.8527±0.0659 (0.6881, 0.9434) | | |
| **Relation extraction** | |  | |  | | |  | | |  | | | |  | | | |  | | | |  | | |  | | | |  | | | |  | |  | | |  | |
| ChemProt | Macro F1 | 0.2127±0.1024 (0.0535, 0.4070) | | | 0.2864±0.1063 (0.1032, 0.4904) | | | 0.1159±0.0881 (0.0130, 0.3290) | | | | 0.2041±0.0962 (0.0605, 0.4041) | | | | 0.3066±0.1172 (0.1037, 0.5241) | | | | 0.0499±0.0601 (0.0000, 0.1901) | | | | 0.1965±0.0934 (0.0747, 0.3948) | | | | 0.3427±0.1284 (0.1315, 0.6191) | | | | 0.0855±0.0685 (0.0139, 0.2693) | | | 0.4264±0.1735 (0.1689, 0.8336) | | 0.3612±0.1555 (0.1390, 0.6967) | | |
| DDI2013 | Macro F1 | 0.2161±0.1022 (0.0661, 0.4674) | | | 0.2653±0.0943 (0.0991, 0.4523) | | | 0.1038±0.0811 (0.0157, 0.3060) | | | | 0.1718±0.0869 (0.0342, 0.3800) | | | | 0.2905±0.0936 (0.1240, 0.4717) | | | | 0.1886±0.0758 (0.0888, 0.3972) | | | | 0.1738±0.0896 (0.0553, 0.4092) | | | | 0.2852±0.0991 (0.1139, 0.4810) | | | | 0.2182±0.0713 (0.1231, 0.3948) | | | 0.5427±0.1661 (0.2263, 0.9031) | | 0.5214±0.1706 (0.2190, 0.8612) | | |
| **Multi-label document classification** | | | | |  | |  | |  | | | |  | | | |  | | | |  | | |  | |  | | | |  | | | |  |  | | | |  |
| HoC | Macro F1 | 0.6013±0.0811 (0.4586, 0.7522) | | | 0.6608±0.0705 (0.5160, 0.7777) | | | 0.1125±0.0523 (0.0224, 0.2221) | | | | 0.6055±0.0868 (0.4405, 0.7690) | | | | 0.6573±0.0712 (0.5128, 0.7863) | | | | 0.2823±0.0561 (0.1722, 0.3951) | | | | 0.6448±0.0809 (0.4772, 0.7836) | | | | 0.6626±0.0682 (0.5051, 0.7846) | | | | 0.1698±0.0337 (0.1068, 0.2252) | | | 0.6171±0.0737 (0.4586, 0.7515) | | 0.3802±0.0816 (0.2458, 0.5279) | | |
| LitCovid | Macro F1 | 0.5625±0.0686 (0.4201, 0.6733) | | | 0.5533±0.0681 (0.4265, 0.6817) | | | 0.3717±0.0298 (0.3164, 0.4257) | | | | 0.5609±0.0693 (0.4227, 0.6797) | | | | 0.5545±0.0691 (0.4318, 0.6899) | | | | 0.4605±0.0669 (0.3321, 0.5995) | | | | 0.5816±0.0582 (0.4678, 0.6728) | | | | 0.5764±0.0646 (0.4554, 0.6928) | | | | 0.3088±0.0584 (0.2054, 0.4281) | | | 0.5201±0.0769 (0.4006, 0.6690) | | 0.4139±0.0455 (0.3497, 0.5124) | | |
| **Question answering** | |  | |  | | |  | | |  | | | |  | | | |  | | | |  | | |  | | | |  | | | |  | |  | | |  | |
| MedQA (5-Option) | Accuracy | 0.4957±0.1081 (0.3000, 0.7000) | | | 0.7077±0.0751 (0.5667, 0.8667) | | | 0.2470±0.0827 (0.1000, 0.4000) | | | | 0.5050±0.1014 (0.3333, 0.7175) | | | | 0.7297±0.0861 (0.5667, 0.9000) | | | | 0.2740±0.0844 (0.1000,0.4175) | | | | 0.5163±0.0993 (0.3333, 0.7175) | | | | 0.7593±0.0798 (0.5825, 0.9000) | | | | 0.3420±0.0845 (0.1667, 0.4842) | | | 0.4400±0.0886 (0.2825, 0.6333) | | 0.3910±0.0818 (0.2333, 0.5333) | | |
| PubMedQA | Accuracy | 0.6693±0.0871 (0.5000, 0.8333) | | | 0.6353±0.0855 (0.4825, 0.7842) | | | 0.5673±0.0825 (0.4158, 0.7333) | | | | 0.4590±0.0870 (0.2825, 0.6508) | | | | 0.7097±0.0862 (0.5158, 0.8508) | | | | 0.2663±0.0728 (0.1333, 0.4333) | | | | 0.6880±0.0876 (0.5000, 0.8333) | | | | 0.7430±0.0744 (0.6000, 0.8667) | | | | 0.5870±0.0794 (0.4333, 0.7333) | | | 0.7990±0.0664 (0.6667, 0.9175) | | 0.7727±0.0671 (0.6158, 0.9000) | | |
| **Text summarization** | |  | |  | | |  | | |  | | | |  | | | |  | | | |  | | |  | | | |  | | | |  | |  | | |  | |
| PubMed | Rouge-L | 0.2278±0.0126 (0.2086, 0.2570) | | | 0.2402±0.0139 (0.2184, 0.2714) | | | 0.1209±0.0135 (0.0991, 0.1538) | | | | 0.2349±0.0117 (0.2152, 0.2600) | | | | 0.2426±0.0143 (0.2182, 0.2751) | | | | 0.0991±0.0027 (0.0937, 0.1040) | | | | 0.2410±0.0137 (0.2153, 0.2679) | | | | 0.2440±0.0144 (0.2221, 0.2790) | | | | 0.1635±0.0119 (0.1445, 0.1881) | | | 0.1889±0.0241 (0.1512, 0.2362) | | 0.1693±0.0165 (0.1428, 0.2099) | | |
| MS^2 | Rouge-L | 0.0891±0.0093 (0.0736, 0.1068) | | | 0.1225±0.0074 (0.1097, 0.1363) | | | 0.0963±0.0081 (0.0825, 0.1119) | | | | 0.1132±0.0089 (0.0963, 0.1308) | | | | 0.1244±0.0075 (0.1092, 0.1392) | | | | 0.0324±0.0035 (0.0262, 0.0396) | | | | 0.1015±0.0093 (0.0836, 0.1215) | | | | 0.1217±0.0077 (0.1078, 0.1360) | | | | 0.1218±0.0091 (0.1028, 0.1389) | | | 0.0936±0.0115 (0.0741, 0.1162) | | 0.0058±0.0056 (0.0000, 0.0197) | | |
| **Text simplification** | |  | |  | | |  | | |  | | | |  | | | |  | | | |  | | |  | | | |  | | | |  | |  | | |  | |
| Cochrane | Rouge-L | 0.2361±0.0124 (0.2168, 0.2676) | | | 0.2364±0.0119 (0.2154, 0.2617) | | | 0.2073±0.0127 (0.1868, 0.2354) | | | | 0.2434±0.0130 (0.2215, 0.2730) | | | | 0.2380±0.0107 (0.2211, 0.2608) | | | | 0.2175±0.0144 (0.1922, 0.2469) | | | | 0.2467±0.0130 (0.2244, 0.2771) | | | | 0.2459±0.0126 (0.2249, 0.2742) | | | | 0.2279±0.0147 (0.2027, 0.2559) | | | 0.2315±0.0173 (0.2044, 0.2633) | | 0.2347±0.0174 (0.2052, 0.2727) | | |
| PLOS | Rouge-L | 0.2317±0.0091 (0.2166, 0.2483) | | | 0.2262±0.0081 (0.2109, 0.2409) | | | 0.2118±0.0099 (0.1958, 0.2292) | | | | 0.2456±0.0095 (0.2287, 0.2630) | | | | 0.2387±0.0087 (0.2242, 0.2554) | | | | 0.1832±0.0150 (0.1512, 0.2108) | | | | 0.2423±0.0096 (0.2235, 0.2579) | | | | 0.2420±0.0094 (0.2241, 0.2610) | | | | 0.1664±0.0091 (0.1492, 0.1867) | | | 0.2581±0.0146 (0.2336, 0.2833) | | 0.2579±0.0143 (0.2336, 0.2887) | | |

Table S2 further shows the performance mean, variance, and confidence intervals of the primary metrics. The results are obtained using bootstrapping with a subsample size of 30 and repeated the process 100 times at a 95% confidence interval*.*

**S2.4 Statistic test results**

Supplementary Data 1 provides the detailed statistic test results to quantify the statistical difference between the LLM performance on the 12 datasets.

**S2.5. Dynamic K-nearest few-shot results**

Table S3. Detailed dynamic K-nearest few-shot results.

|  |  | GPT-3.5 | | | GPT-4 | | | LLaMA2 13B | | | | | |
| --- | --- | --- | --- | --- | --- | --- | --- | --- | --- | --- | --- | --- | --- |
|  |  | K = 1 shot | 2 shot | 5 shot | 1 shot | 2 shot | 5 shot | 1 shot | 2 shot | 5 shot |  |  |  |
| **Named entity recognition** | |  |  |  |  |  |  |  |  |  |  |  |  |
| BC5CDR-chemical | Entity F1 | 0.6728 | 0.6738 | 0.6787 | 0.7904 | 0.7959 | 0.7950 | 0.5529 | 0.6184 | 0.6922 |  |  |  |
| NCBI Disease | Entity F1 | 0.4154 | 0.4093 | 0.3769 | 0.5262 | 0.4980 | 0.4881 | 0.4696 | 0.5466 | 0.5897 |  |  |  |
| **Relation extraction** | |  |  |  |  |  |  |  |  |  |  |  |  |
| ChemProt | Macro F1 | 0.1419 | 0.1455 | 0.1463 | 0.2205 | 0.3353 | 0.3435 | 0.0970 | 0.1477 | 0.1206 |  |  |  |
|  | Micro F1 | 0.2408 | 0.1824 | 0.1820 | 0.4396 | 0.5092 | 0.5275 | 0.1505 | 0.7055 | 0.2195 |  |  |  |
| DDI2013 | Macro F1 | 0.1229 | 0.2419 | 0.2078 | 0.2965 | 0.2936 | 0.3175 | 0.1316 | 0.1460 | 0.2506 |  |  |  |
|  | Micro F1 | 0.3281 | 0.3508 | 0.3822 | 0.5206 | 0.5020 | 0.5248 | 0.1701 | 0.2625 | 0.5353 |  |  |  |
| **Multi-label document classification** | | |  |  |  |  |  |  |  |  |  |  |  |
| HoC | Macro F1 | 0.6931 | 0.6885 | 0.7242 | 0.7177 | 0.7035 | 0.7322 | 0.2792 | 0.2990 | 0.3745 |  |  |  |
|  | Micro F1 | 0.6866 | 0.6885 | 0.7290 | 0.7207 | 0.7105 | 0.7330 | 0.3334 | 0.3340 | 0.4209 |  |  |  |
| LitCovid | Macro F1 | 0.6364 | 0.6357 | 0.6484 | 0.6500 | 0.6633 | 0.7055 | 0.5254 | 0.5352 | 0.4607 |  |  |  |
|  | Micro F1 | 0.7048 | 0.7056 | 0.7229 | 0.7359 | 0.7546 | 0.7927 | 0.6460 | 0.6526 | 0.5991 |  |  |  |
| **Question answering** | |  |  |  |  |  |  |  |  |  |  |  |  |
| MedQA (5-Option) | Accuracy | 0.5192 | 0.5153 | 0.5295 | 0.7376 | 0.7596 | 0.7753 | 0.3378 | 0.3621 | 0.3291 |  |  |  |
|  | Macro F1 | 0.4311 | 0.4280 | 0.4397 | 0.6143 | 0.6328 | 0.6468 | 0.2384 | 0.2603 | 0.2794 |  |  |  |
| PubMedQA | Accuracy | 0.5740 | 0.5740 | 0.6500 | 0.7200 | 0.7420 | 0.7560 | 0.3840 | 0.2120 | 0.3460 |  |  |  |
|  | Macro F1 | 0.5073 | 0.4964 | 0.5242 | 0.5728 | 0.5851 | 0.5836 | 0.3696 | 0.2182 | 0.2581 |  |  |  |
| **Text summarization** | |  |  |  |  |  |  |  | | | | | |
| PubMed | Rouge-L | 0.2472 | 0.2482 | 0.2438 | 0.2524 | 0.2538 | 0.2411 | 0.1969 | 0.1852 | 0.1746 | |  |  |
|  | BERT score | 0.7267 | 0.7278 | 0.7251 | 0.7273 | 0.7271 | 0.7200 | 0.7017 | 0.6970 | 0.6881 | |  |  |
|  | BART score | -4.7751 | -4.7609 | -4.7920 | -4.8142 | -4.7919 | -4.8697 | -4.7869 | -4.6472 | -4.7485 | |  |  |
| MS^2 | Rouge-L | 0.0974 | 0.0946 | 0.0946 | 0.1230 | 0.1234 | 0.1226 | 0.0983 | 0.1015 | 0.1025 | |  |  |
|  | BERT score | 0.6681 | 0.6660 | 0.6654 | 0.6879 | 0.6877 | 0.6868 | 0.6377 | 0.6319 | 0.6281 | |  |  |
|  | BART score | -5.1329 | -5.1411 | -5.1428 | -5.2626 | -5.2633 | -5.2838 | -5.6137 | -5.6230 | -5.6402 | |  |  |
| **Text simplification** | |  | | | | | | | | | | |  |
| Cochrane | Rouge-L | 0.2528 | 0.2538 | 0.2536 | 0.2567 | 0.2587 | 0.2587 | 0.2260 | 0.2339 | 0.2295 | | |  |
|  | FKG | 12.3898 | 12.4502 | 12.5908 | 11.8360 | 11.6763 | 11.8388 | 12.0290 | 11.7515 | 12.5125 | | |  |
|  | DCR | 9.7913 | 9.8791 | 9.9214 | 9.5561 | 9.4289 | 9.4811 | 10.0548 | 10.0906 | 9.8953 | | |  |
| PLOS | Rouge-L | 0.2451 | 0.2466 | 0.2482 | 0.2430 | 0.2416 | 0.2430 | 0.2330 | 0.2392 | 0.1790 | | |  |
|  | FKG | 13.2760 | 13.2652 | 13.3785 | 12.1148 | 12.0537 | 12.0698 | 14.1853 | 14.2004 | 16.5514 | | |  |
|  | DCR | 10.8511 | 10.7889 | 10.7330 | 10.1539 | 10.0404 | 10.0848 | 10.9964 | 10.8053 | 9.0939 | | |  |

Table S3 shows the detailed dynamic K-nearest few-shot results.

**S3. Qualitative evaluation on the PubMed Text Summarization Benchmark**

**S3.1 Annotation guideline**

For each model response, please rate the following. Keep in mind that the order of the model outputs may be randomly shuffled, meaning that what's Model 1 in sample 1 could become Model 2 in sample 2 as an example.

Rating ranges from 1 (bad) 2,3,4 to 5 (good):

**1. Accuracy of generated summaries: Does the summary contain correct information from the original article?**

1 (bad): The summary includes false or misleading information that is significantly different from the original article.

2: Some elements of the summary contain correct information however the overall summary is inaccurate.

3: The main point of the summary is correct, however it may include some inaccurate information from the original article.

4: The summary mostly avoids inaccuracies, but may include minor inaccurate information from the original article.

5 (good): The summary is accurate based on the original article.

Additional annotations: if the response contains false or misleading information, please identify them.

**2. Completeness of generated summaries: Does the summary capture the key information from the original article?**

1 (bad): The summary is incomplete (missing key information), or leaves out crucial details.

2: The summary is somewhat complete, but it lacks key information that impact its comprehensiveness.

3: The summary is moderately complete, but certain details are missing, requiring enhancement.

4: The summary is largely comprehensive, but a few minor details could be refined for better alignment with the original article.

5 (good): The summary text is comprehensive and includes all relevant information.

**3. Readability of generated summaries: Is the summary easy to read?**

1 (bad): The text is highly difficult to read, full of grammatical errors, and lacks coherence and clarity.

2: The text is somewhat difficult to read, and there are occasional grammatical errors. The coherence and clarity could be improved.

3: The text is moderately easy to read, but there are noticeable grammatical errors and some parts lack coherence and clarity.

4: The text is fairly easy to read, with only a few minor grammatical errors. Overall coherence and clarity are good, but there is room for improvement.

5 (good): The text is easy to read, well-structured, and flows naturally.

**S3.2 Statistic test results**

The **qualitative_evaluation_summarization_statistic_test.xlsx** file provides the detailed statistic test results.

**S3.3 Examples**

**Example 1**

**1.1 Gold standard:**

purposethis study was aimed to determine the effect of two chemically distinct denture cleansers and water on the surface hardness of acrylic and silicone based soft denture liners at various time intervals.materials and methodstwo commonly used commercial resilient liner material were selected based on their chemical composition ( silicone- and acrylic - based soft liners ) for this investigation .
 120 cylindrical specimens were made of 15 mm 10 mm dimensions ( according to astm : d-2240 - 64 t ) in a custom made metal mold .
 all specimens were stored in artificial saliva throughout the study .
 forty specimens were cleansed daily in 0.5% sodium hypochlorite solution ; forty were cleansed in sodium perborate and remaining forty specimens were daily rinsed in water .
 testing was done at 1 week , 1 month , 3 months and 6 months for surface hardness using a shore a durometer .
 a mean of 3 reading for each sample was subjected to one - way anova , post hoc test and pair - t test for statistical analysis .
 p values of less than 0.05 were taken as statistically significant.resultssurface hardness of all the samples was significantly higher after a period of 6 months irrespective of the cleansing treatment .
 minor changes were observed between control , sodium hypochlorite and sodium perborate groups with time .
 greater change was observed in surface hardness of acrylic - based soft denture liners as compared to silicone - based soft liners for all groups , as time progressed.conclusionsilicone-based soft denture liners performed significantly better in all cleansing treatments than acrylic - based soft denture liners .

**1.2 Fine-tuned BART:**

**Annotations: Accuracy: 4.5; Completeness: 3; Readability: 3**

purposethe aim of this study was to determine the effect of two chemically distinct denture cleansers and water on the surface hardness of acrylic and silicone based soft denture liners at various time intervals.materials and methodsthe study was conducted in maharishi markandeshwar college of dental sciences and research , mullana , ambala , haryana , india .

two commonly used commercial resilient liner materials based on their chemical composition ( silicone- and acrylic - based soft liners ) were selected for the study .

a total of hundred and twenty cylindrical specimens were made of the dimensions 15 mm 10 mm ( according to astm : d-2240 - 64t ) .

soft denture liners were manipulated according to manufacturer 's instructions and expressed into the mold .

the mold was then covered from the top by a cellophane sheet and another glass slab was pressed tightly against the mold to remove excess material and to shape the specimens according to the dimensions of the mold .

group a ( control ) , group b ( sodium hypochlorite ) and group c ( sodium perborate ) comprising of 40 specimens each . each group was divided further into 4 subgroups : subgroup i , ii , iii and iv ( consisted of 10 specimens each ) to be tested at a time interval of 1 week , 1 month , 3 months and 6 months respectively .

each subgroup was again divided into two minor subgroups : minor subgroup a - consisted samples made of acrylic based soft denture liner and minor subgroup b - consisted of samples made of silicone based soft denture liner .

specimens in group a ( control ) were cleansed daily by rinsing with water and then were stored in artificial saliva for the entire period of the study .

specimens in group b were immersed in 0.5% sodium hypochlorite solution for ten minutes daily , rinsed in water and stored in artificial saliva at room temperature .

specimens in group c were cleansed in a solution of sodium perborate denture cleansing tablets ( dissolved in 250 ml water as recommended by the manufacturer ) .

specimens of all 3 groups were tested at 1 week , 1 month and 3 months time intervals at central institute of plastics engineering & technology ( cipet ) , panipat , haryana.resultsthe specimens were tested using a shore a durometer which

**1.3 GPT-3.5 zero-shot**

**Annotations: Accuracy: 4.5; Completeness: 3.5; Readability: 5**

soft denture lining materials, such as acrylic and silicone types, are useful for treating patients with ridge atrophy or resorption, bony undercuts, bruxing tendencies, congenital or acquired oral defects, xerostomia, and dentures opposing natural dentition. however, these materials present problems such as weakening of bond, loss of resiliency, color alterations, and porosity. rougher surfaces of the lining materials can enhance adhesion of microorganisms and fungal growth, so regular cleansing procedures are necessary to prevent infection. mechanical cleansing is not advisable for soft denture liners, so chemical cleansing using denture cleansers is the preferred method. alkaline peroxide and sodium hypochlorite cleansers are commonly used, with sodium perborate being the most effective. in a study comparing acrylic and silicone-based soft denture liners, it was found that silicone-based liners performed better in terms of surface hardness over a period of 6 months when cleansed daily. chemical denture cleansers can be used daily without affecting the surface hardness of soft denture liners, and silicone-based liners showed better compatibility with cleansing solutions and maintained their resiliency better.

**1.4 GPT-4 zero-shot**

**Annotations: Accuracy: 5; Completeness: 3.5; Readability: 5**

the study investigates the effect of two chemically distinct denture cleansers on the surface hardness of acrylic and silicone based soft denture liners. soft denture liners are used to make wearing dentures more comfortable for patients, but can present problems such as weakening of bond between lining and denture, loss of resiliency, and increased surface roughness. denture cleansers are essential for maintaining oral hygiene, with peroxide cleansers being the most commonly used. the study found that after one month, significant changes were noted in surface hardness for acrylic samples when compared to those of sodium hypochlorite group and sodium perborate group. at six months, silicone-based soft denture liners showed better compatibility with cleansing solutions and maintained their resiliency better, proving to be more promising for long term usage. the study concludes that chemical denture cleansers can be used daily to cleanse soft denture liners without adversely affecting their surface hardness.

**1.5 LLaMA 2 13B zero-shot**

**Annotations: Accuracy: 3.5; Completeness: 2; Readability: 1.5**

acrylic - based soft denture liners at all time intervals . for silicone - based soft denture liners highly significant increase in surface hardness was seen at 1 month and 3 months . in sodium perborate group , highly significant increase in surface hardness was seen for acrylic - based soft denture liners at all time intervals . for silicone - based soft denture liners highly significant increase in surface hardness was seen at 1 month and 3 months . the results of the present study indicate that silicone - based soft denture liners are more resistant to the effects of denture cleansers than acrylic - based soft denture liners . the results of the present study are in agreement with the findings of mese and guzel5 who concluded that silicone - based soft denture liners are more resistant to the effects of denture cleansers than acrylic - based soft denture liners . the results of the present study are also in agreement with the findings of sara et al.14 who concluded that sodium perborate denture cleanser proved to be most effective . the results of the present study are also in agreement with the findings of de freitas fernandes et al.15 and ferreira et al.16 who concluded that the best results were found for the treatment with 0.5% naocl for 10 minutes in comparison to other cleansers . the results of the present study are also in agreement with the findings of mese and guzel5 who concluded that after 6 months hardness values of all resilient liners evaluated were higher with increased duration of immersion . the hardness values of acrylic resin - based liners showed greater change than those of silicone products . the results of the present study are also in agreement with the findings of mese and guzel5 who concluded that the hardness values of acrylic resin - based liners showed greater change than those of silicene products . the results of the present study are also in agreement with the findings of mese and guzel5 who concluded that the hardness values of acrylic resin - based liners showed greater change than those of silicene products . the results of the present study are also in agreement with the findings of mese and guzel5 who concluded that

**Example 2**

**2.1 Gold standard**

an inflammatory myofibroblastic tumor ( imt ) is a distinctive neoplasm composed of myofibroblastic and fibroblastic spindle cells , accompanied by inflammatory infiltration of plasma cells , lymphocytes , and eosinophils .

imts rarely occur in the urinary bladder .

it is important to distinguish this tumor from other malignant spindle cell tumors .

herein , we report a patient with an imt showing muscle invasion , who underwent a transurethral resection of the bladder tumor and , at a later date , partial cystectomy . the resected tumor specimen revealed a proliferation of spindle - shaped cells on a background of plasma cells and lymphocytes .

immunohistochemical staining showed the tumor to be positive for anaplastic lymphoma kinase ( alk ) , smooth muscle actin , and vascular endothelial growth factor ( vegf ) .

such histopathological findings were indicative of an imt , suggesting the use of inhibitors of alk and vegf as pharmacotherapy .

**2.2 Fine-tuned BART**

**Annotations: Accuracy: 5; Completeness: 4.5; Readability: 4.5**

an inflammatory myofibroblastic tumor ( imt ) is a distinctive neoplasm composed of myofibroblastic and fibroblastic spindle cells accompanied by inflammatory infiltration of plasma cells , lymphocytes , and eosinophils .

it is important to distinguish this tumor from other malignant spindle cell tumors , such as the sarcomatoid variant of urothelial carcinoma and leiomyosarcoma .

we report a case of imt of the urinary bladder in a 52-year - old male diagnosed by transurethral resection of the bladder tumor ( turbt ) treated by partial cystectomy .

histopathology results were consistent with an imt .

the bladder tissue , including muscle , was widely infiltrated by spindle cells in a myxoid stroma accompanied by infiltration of inflammatory cells .

immunohistochemical staining was positive for vimentin and smooth muscle actin and negative for ae1/ae3 , desmin , myogenin , s-100 , cd34 , c - kit , cd68 , and bcl-2 .

a pathological examination of the excised specimen revealed the proliferation of spindle cells expanding into the bladder musculature , accompanied by inflammatory cell infiltration .

for the assessment of therapeutic options , we measured the expressions of anaplastic lymphoma kinase ( alk ) , vascular endothelial growth factor ( vegf ) , and cyclooxygenase 2 ( cox2 ) , known as growth factors involved in tumor proliferation .

**2.3 GPT-3.5 zero-shot**

**Annotations: Accuracy: 5; Completeness: 3; Readability: 4.5**

this case report describes a 52-year-old male with an inflammatory myofibroblastic tumor (imt) of the urinary bladder. the patient had a history of rheumatoid arthritis and was treated with prednisolone. imaging studies revealed a bladder tumor infiltrating the muscle layer. the tumor was resected by partial cystectomy, and histopathology confirmed the diagnosis of imt. immunohistochemical staining showed positive expression of vimentin and smooth muscle actin, and negative expression of other markers. the expression of growth factors involved in tumor proliferation, such as anaplastic lymphoma kinase (alk), vascular endothelial growth factor (vegf), and cyclooxygenase 2 (cox2), was also measured. surgical resection is the main treatment for imt, but pharmacotherapy may be considered for nonresectable tumors. close follow-up is recommended for imt cases.

**2.4 GPT-4 zero-shot**

**Annotations: Accuracy: 5; Completeness: 3.5; Readability: 4.5**

an inflammatory myofibroblastic tumor (imt) is a unique neoplasm composed of myofibroblastic and fibroblastic spindle cells, often accompanied by inflammatory infiltration. a case of imt in the urinary bladder of a 52-year-old male was diagnosed and treated by partial cystectomy. the tumor was characterized by atypical spindle cell proliferation and inflammatory cell infiltrates, primarily involving lymphocytes and plasma cells. imts are classified as intermediate tumors and are often found in the mesentery, omentum, retroperitoneum, pelvis, and abdominal soft tissues. however, occurrence in the urinary bladder is unusual. in terms of treatment, surgical resection is often used, but pharmacotherapy may become a viable therapeutic option in cases of recurrence when the tumor is not resectable.

**2.5 LLaMA 13B zero-shot**

**Annotations: Accuracy: 5; Completeness: 3; Readability: 3**

an inflammatory myofibroblastic tumor ( imt ) is a distinctive neoplasm composed of myofibroblastic and fibroblastic spindle cells accompanied by inflammatory infiltration of plasma cells , lymphocytes , and eosinophils . it is important to distinguish this tumor from other malignant spindle cell tumors , such as the sarcomatoid variant of urothelial carcinoma and leiomyosarcoma . we report a case of imt of the urinary bladder in a 52 - year - old male diagnosed by transurethral resection of the bladder tumor ( turbt ) treated by partial cystectomy . his medical history revealed a diagnosis of rheumatoid arthritis 3 years ago , and prednisolone 10 mg / day was subsequently prescribed . cystoscopy revealed a solitary nonpapillary tumor with surrounding edema at the dome of the bladder . enhanced computed tomography and magnetic resonance imaging revealed an early enhancing bladder tumor infiltrating the outside of the bladder muscle layer . intraoperative findings showed a large solid bladder mass , measuring approximately 3 cm from the dome to the anterior wall of the bladder . we resected the tumor to the depth of the muscle layer , but normal muscle tissue was not seen . histopathology results were consistent with an imt . the bladder tissue , including muscle , was widely infiltrated by spindle cells in a myxoid stroma accompanied by infiltration of inflammatory cells . immunohistochemical staining was positive for vimentin and smooth muscle actin and negative for ae1/ae3 , desmin , myogenin , s - 100 , cd34 , c - kit , cd68 , and bcl - 2 . the excised specimen is shown in figure 2d . a pathological examination of the excised specimen revealed the proliferation of spindle cells expanding into the bladder musculature , accompanied by inflammatory cell infiltration . for

**Example 3**

**3.1 Gold standard**

backgroundit is recognized that candida dubliniensis commonly colonizes oral and subgingival sites in immunocompetent subjects with periodontal disease.objectivesince there are few data available on genetic characterization of c. dubliniensis in periodontal pockets and other oral sites , the aim of this study was to characterize subgingival and mucosal c. dubliniensis isolates recovered from immunocompetent subjects and to assay the genetic similarity of such isolates from both niches in the same patient by random amplified polymorphic dna ( rapd).design

c. dubliniensis recovered from subgingival plaque and from buccal cavity samples were studied in 240 immunocompetent non - smoking individuals . arbitrary amplification was carried out by rapd - polymerase chain reaction ( pcr).resultsrapd analysis showed identical genotypes of c. dubliniensis in different sampling sites ( buccal cavity and subgingival areas ) in eight of 10 patients except for those derived from two participants who presented presumably unrelated isolates.conclusionson the basis of the findings presented , the origin of the colonization of c. dubliniensis in subgingival biofilm seems to be the buccal cavity in a single patient .

consequently , it may be assumed that most of c. dubliniensis in these sites arise from the endogenous commensal strains .

**3.2 Fine-tuned BART**

**Annotations: Accuracy: 5; Completeness: 4; Readability: 4.5**

background : candida dubliniensis is a yeast species that is characterized by its in vitro resistance to the antifungal azole group in hiv patients , for their capacity to adhere in vitro to human buccal epithelial cells and other microorganisms in the oral microbiota , as well as the high proteinase activity.objectives:the aim of this study was to determine the prevalence of c. dubliniensis in the buccal cavity and subgingival biofilm in immunocompetent individuals.materials and methods : a total of 240 individuals were included in the study .

clinical periodontal evaluations included clinical examination and radiographs with clinical measurements : clinical attachment level ( cal ) , pocket depth ( pd ) , plaque index ( 29 ) , gingival index ( 30 ) , and bleeding on probing ( bop ) .

yeasts were isolated by conventional mycological methods : colony color on the chromogenic medium , micromorphology in agar milk with 1% tween 80 , and carbohydrate assimilation tests using the commercially available kit api i d 32c .

dendrogram was generated by the unweighted pair group method using arithmetic averages ( upgma).results : out of the 203 yeasts , c. dubliniensis was isolated in 4.2% ( 10/240 ) and 4.6% ( 11/240 ) of patients in both niches , respectively .

eight genetic clusters and five main genotypes were obtained at an sc of 90% , genotypes i , ii , iii , iv , and v.conclusions:c. dubliniensis can colonize subgingival biofilm in both healthy and immunocompetent individuals .

**3.3 GPT-3.5 zero-shot**

**Annotations: Accuracy: 5; Completeness: 4.5; Readability: 5**

a study was conducted to investigate the presence and distribution of candida dubliniensis in the buccal cavity and subgingival biofilm of individuals with periodontal disease. a total of 21 isolates of c. dubliniensis were collected from 240 non-smoking individuals. clinical evaluations were performed, and measurements of periodontal parameters were taken. the study found that c. dubliniensis was present in both the buccal cavity and subgingival biofilm, with a prevalence of 4.2% and 4.6% respectively. the species distribution of yeast isolates varied according to the periodontal health status. rapd analysis showed that c. dubliniensis isolates had similar genotypes in different sampling sites, suggesting a common clonal origin. these findings provide baseline information on the colonization of c. dubliniensis in the oral cavity.

**3.4 GPT-4 zero-shot**

**Annotations: Accuracy: 5; Completeness: 4; Readability: 4.5**

the study collected 21 isolates of c. dubliniensis from 240 non-smoking individuals at the university of buenos aires dental clinic. the samples were taken from the buccal cavity and subgingival biofilm, and were cultured on a differential chromogenic medium. the isolated yeasts were identified using conventional mycological methods and further characterized using specific pcr with primers from actin gene-associated intron sequences of c. dubliniensis. the study also used rapd-pcr analysis for genetic characterization of the isolates. the results showed that c. dubliniensis was isolated in 4.2% and 4.6% of patients in the buccal cavity and subgingival biofilm respectively. the study concluded that the origin of the colonization of c. dubliniensis in subgingival biofilm seems to be the buccal cavity.

**3.5 LLaMA 2 13B zero-shot**

**Annotations: Accuracy: 1.5; Completeness: 1; Readability: 1**

. 1 ) . the other case was patient 10 , who presented two genotypes in the buccal cavity and three in the subgingival biofilm . the genotypes of c. dubliniensis in the buccal cavity and subgingival biofilm were identical in 80% of the patients . the genotypes of c. dubliniensis in the buccal cavity and subgingival biofilm were identical in 80% of the patients . the genotypes of c. dubliniensis in the buccal cavity and subgingival biofilm were identical in 80% of the patients . the genotypes of c. dubliniensis in the buccal cavity and subgingival biofilm were identical in 80% of the patients . the genotypes of c. dubliniensis in the buccal cavity and subgingival biofilm were identical in 80% of the patients . the genotypes of c. dubliniensis in the buccal cavity and subgingival biofilm were identical in 80% of the patients . the genotypes of c. dubliniensis in the buccal cavity and subgingival biofilm were identical in 80% of the patients . the genotypes of c. dublininensis in the buccal cavity and subgingival biofilm were identical in 80% of the patients . the genotypes of c. dublininensis in the buccal cavity and subgingival biofilm were identical in 80% of the patients . the genotypes of c. dublininensis in the buccal cavity and subgingival biofilm were identical in 80% of the patients . the genotypes of c. dublininensis in the buccal cavity and subgingival biofilm were identical in 80% of the patients . the genotypes of c. dublininensis in the buccal cavity and subgingival biofilm were identical in 80% of the patients . the genotypes of c. dublininensis in the buccal cavity and subgingival biofilm were identical in 80% of the patients . the genotypes of c. dublininensis in the buccal cavity and sub
