# Supplementary material for: Benchmarking large language models for biomedical natural language processing applications and recommendations
Source: Nat Commun. 2025 Apr 6;16:3280. doi: 10.1038/s41467-025-56989-2 (PMC11972378; doi:10.1038/s41467-025-56989-2)
Supplement: Supplementary file 3 — Description of Additional Supplementary Files [file 41467_2025_56989_MOESM3_ESM.pdf]

Supplementary Data 1. The detailed statistical comparison for every model pair per dataset, in addition to Table 3. For each dataset, we computed the statistical results for every possible model pair using the two-sided Wilcoxon rank-sum test mentioned in the Evaluation section of the main manuscript and S2.1 in the Supplementary Information.
