# Supplementary material for: Benchmarking large language models for biomedical natural language processing applications and recommendations
Source: Nat Commun. 2025 Apr 6;16:3280. doi: 10.1038/s41467-025-56989-2 (PMC11972378; doi:10.1038/s41467-025-56989-2)
Supplement: Supplementary file 4 — Supplementary Data 1 [file 41467_2025_56989_MOESM4_ESM.zip › quantitative_evaluation_results/readme.docx]

The qualitative evaluation results folder contains the detailed statistical comparison for every model pair per dataset in addition to Table 3. For each dataset, we computed the statistical results every possible model pairs using the two-sided Wilcoxon rank-sum test mentioned in the Evaluation section of the main manuscript and S2.1 in the supplementary material.

The results per dataset are summarized in each spreadsheet. Each row in the spreadsheet contains the statistical results of a model pair. The model name is structured as [dataset]_[model]_[setting]. The setting consists of zs for zero-shot, os for one-shot, 5s for five-shot, and finetuned for fine-tuning. For instance, BC5CDR_Chemical_gpt3.5_zs is GPT-3.5 on the BC5CDR Chemical dataset under zero-shot. The statistical results consists of means, standard deviations, confidence internals, and P-values.
